# Shortened Tracer Uptake Time in GA-68-DOTATOC-PET of Meningiomas Does Not Impair Diagnostic Accuracy and PET Volume Definition

**DOI:** 10.3390/diagnostics10121084

**Published:** 2020-12-13

**Authors:** Josefine Graef, Carolin Senger, Christoph Wetz, Alexander D. J. Baur, Anne K. Kluge, Mathias Lukas, Julian M. M. Rogasch, Thula C. Walter-Rittel, David Kohnert, Marcus Makowski, Güliz Acker, Kai Huang, Volker Budach, Holger Amthauer, Imke Schatka, Christian Furth

**Affiliations:** 1 Department of Nuclear Medicine, Charité-Universitätsmedizin Berlin, Corporate Member of Freie Universität Berlin, Humboldt-Universität zu Berlin, and Berlin Institute of Health, 13353 Berlin, Germany; christoph.wetz@charite.de (C.W.); alexander.baur@charite.de (A.D.J.B.); julian.rogasch@charite.de (J.M.M.R.); thula.walter-rittel@charite.de (T.C.W.-R.); david.kohnert@charite.de (D.K.); kai.huang@charite.de (K.H.); holger.amthauer@charite.de (H.A.); imke.schatka@charite.de (I.S.); christian.furth@charite.de (C.F.); 2 Department of Radiation Oncology and Radiotherapy, Charité-Universitätsmedizin Berlin, Corporate Member of Freie Universität Berlin, Humboldt-Universität zu Berlin, and Berlin Institute of Health, 13353 Berlin, Germany; carolin.senger@charite.de (C.S.); anne.kluge@charite.de (A.K.K.); volker.budach@charite.de (V.B.); 3 Conradia Charlottenburg MVZ GmbH, 10627 Berlin, Germany; 4 Department of Diagnostic and Interventional Radiology, Universitätsmedizin Leipzig, 04103 Leipzig, Germany; mathias.lukas@medizin.uni-leipzig.de; 5 Department of Diagnostic and Interventional Radiology, Charité-Universitätsmedizin Berlin, Corporate Member of Freie Universität Berlin, Humboldt-Universität zu Berlin, and Berlin Institute of Health,13353 Berlin, Germany; marcus.makowski@tum.de; 6 Department of Diagnostic and Interventional Radiology, School of Medicine, Technical University of Munich, 81675 Munich, Germany; 7 Department of Neurosurgery, Charité-Universitätsmedizin Berlin, Corporate Member of Freie Universität Berlin, Humboldt-Universität zu Berlin, and Berlin Institute of Health, 10117 Berlin, Germany; gueliz.acker@charite.de; 8 Berlin Institute of Health (BIH), Charité University Hospital Berlin and MDC, 10178 Berlin, Germany

**Keywords:** DOTATOC, meningioma, PET, radiosurgery

## Abstract

Ga-68-DOTATOC-PET/MRI can affect the planning target volume (PTV) definition of meningiomas before radiosurgery. A shorter tracer uptake time before image acquisition could allow the examination of more patients. The aim of this study was to investigate if shortening uptake time is possible without compromising diagnostic accuracy and PET volume. Fifteen patients (*f* = 12; mean age 52 years (34–80 years)) with meningiomas were prospectively examined with dynamic [68Ga]Ga-68-labeled [DOTA0-Phe1-Tyr3] octreotide (Ga-68-DOTATOC)-PET/MRI over 70 min before radiosurgery planning. Meningiomas were delineated manually in the PET dataset. PET volumes at each time point were compared to the reference standard 60 min post tracer injection (p.i.) using the Friedman test followed by a Wilcoxon signed-rank test and Bonferroni correction. In all patients, the earliest time point with 100% lesion detection compared to 60 min p.i. was identified. PET volumes did not change significantly from 15 min p.i. (*p* = 1.0) compared to 60 min p.i. The earliest time point with 100% lesion detection in all patients was 10 min p.i. In patients with meningiomas undergoing Ga-68-DOTATOC-PET, the tracer uptake time can safely be reduced to 15 min p.i. with comparable PET volume and 100% lesion detection compared to 60 min p.i.

## 1. Introduction

Meningiomas are the most common intracranial tumors [1]. Treatment is necessary for lesions that become symptomatic due to local tumor growth. Although surgical resection remains the therapy of choice [2], robotic radiosurgery (RRS) has become increasingly attractive as an alternative treatment strategy, especially in patients with residual or recurrent meningiomas or in patients where primary resection is likely to be incomplete or impossible due to complex tumor location, such as at the skull base [3,4,5].

Treatment planning for RRS can be challenging as it requires precise tumor delineation and reliable identification of organs at risk due to irradiation with high single doses without safety margins [6]. Apart from standard diagnostics with contrast-enhanced computed tomography (CT) and magnetic resonance imaging (MRI), nuclear medical diagnostics with positron emission tomography (PET), e.g., as hybrid imaging with MRI (PET/MRI), has become increasingly important in pretherapeutic radiation planning.

The high diagnostic accuracy in the detection of meningiomas of PET with [68Ga]Ga-68-labeled [DOTA0-Phe1-Tyr3] octreotide (Ga-68-DOTATOC) is derived from the high affinity of the somatostatin analog Ga-68-DOTATOC to the somatostatin receptor subtype 2, which is highly expressed in meningiomas [7]. Therefore, the resulting high lesion-to-background ratio enables precise lesion detection and demarcation against the surrounding tissue [8]. As a result, PET/MRI with somatostatin analogs can be used for initial diagnosis, treatment planning (i.e., before resection, radiotherapy, Lu177-DOTATOC/TATE therapy) as well as for follow-up examinations [9,10].

In the case of skull base meningiomas or transosseous manifestation, MRI frequently is not sufficient to differentiate tumor margins from surrounding scar or skull base tissue. In these areas, Ga-68-DOTATOC-PET/MRI can improve the delineation of the planning target volume (PTV) [11]. However, the added value of Ga-68-DOTATOC-PET/MRI-based RRS planning is difficult to achieve due to the limited availability of PET/MRI devices and the short half-life of Ga-68-DOTATOC of 68 min (min). Ga-68-DOTATOC needs to be administered with relatively high activity because 60 min post tracer injection (p.i.), when PET acquisition typically starts, almost half of the injected activity has already decayed. Therefore, a shorter time between tracer injection and image acquisition would be desirable to reduce patients’ waiting time, improve patient throughput and, potentially, to reduce radiation exposure by reducing the administered activity. Given the fact that previous studies have shown high Ga-68-DOTATOC uptake in meningiomas at 10-20 min p.i. [7] and that 80% of the tracer is cleared from the blood pool at 10 min p.i. [12], a shortened uptake time seems feasible. Afshar-Oromieh et al. [13] evaluated patients with meningiomas undergoing Ga-68-DOTATOC-PET/CT 30 min p.i and subsequent PET/MRI 2 h p.i. In the comparison of both exams, meningiomas showed a higher tracer activity concentration and contrast in early images (30 min p.i.). The authors discussed the different detector techniques and different methods of measurements as possible explanations for this observation and finally recommended the implementation of a Ga-68-DOTATOC-PET/MRI 30 min p.i. [13].

Therefore, the aim of this study was to identify the shortest possible tracer uptake time that would enable an identical lesion detection rate as well as a comparable PET volume of meningiomas compared to the standard uptake time 60 min p.i.

## 2. Materials and Methods

### 2.1. Study Design

A total of 15 patients with meningiomas who underwent Ga-68-DOTATOC-PET/MRI as part of their diagnostic imaging before treatment with CyberKnife (CK, Accuray Inc., Sunnyvale, CA, USA) RRS between January 2019 and August 2019 in our department were included in this prospective study. Written informed consent was obtained from all patients. The local ethics committee (vote, EA1/060/16, 18th August 2016) approved the study. 

### 2.2. Ga-68-DOTATOC-PET/MRI

**Figure 1:**
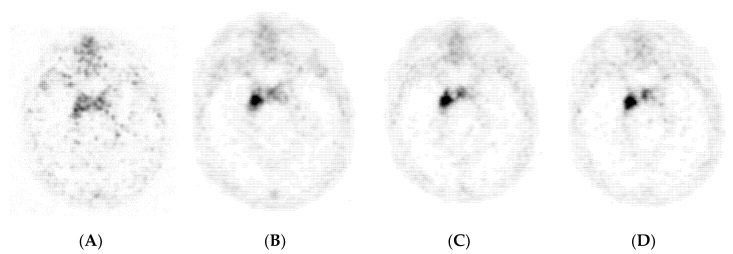
Dynamic PET images of meningioma: (**A**) 1 min (min) post tracer injection (p.i.); (**B**) 5 min p.i.; (**C**) 15 min p.i.; (**D**) 60 min p.i.

PET/MRI scans were performed using a 3T Siemens Biograph mMR (VE11P; Siemens Healthcare GmbH, Erlangen, Germany). After intravenous injection of a median activity of 165.4 MBq (4.47 mCi; range: 120–190 MBq, 3.24–5.14 mCi) Ga-68-DOTATOC, dynamic PET images were obtained for 70 min. In one patient, dynamic acquisition had to be stopped after 60 min p.i. due to imperative urinary urgency. PET data were acquired with a single bed position that covered the entire skull (3D list mode acquisition). Dynamic frames were reconstructed with 6 × 20 s (s), 8 × 1 min and 12 × 5 min for tracer activity concentration evaluation. In addition, 8 different time points p.i. were chosen for PET volume evaluation of meningioma lesions and were reconstructed with 1 × 20 s, 1 × 1 min and 6 × 5 min (image examples, Figure 1). Image reconstruction was performed using ordered-subset expectation maximization (OSEM; 3 iterations, 21 subsets; voxel matrix, 344 × 344 × 127; voxel size, 1.0 × 1.0 × 2.0 mm, no filter). The reconstructed time-dependent data were corrected for patient motion frame by frame. Vendor-provided ultrashort echo-time sequence (UTE) was used for the correction of attenuation and scatter (absolute mode). Simultaneously acquired MR images included T1- and T2-weighted sequences of the entire skull as well as postcontrast (Gadovist; Bayer Vital GmbH; dose: 1.0 mmol/kg body weight) isotropic 3D high-resolution T1-weighted sequences.

### 2.3. Data Analysis

Reconstructed PET data were analyzed using syngo.via (VB 30, Siemens Healthcare GmbH, Erlangen, Germany) and the ECLIPSE^TM^ treatment planning system (Version 15.5, Varian Medical Systems Inc., Palo Alto, CA, USA).

**Figure 2:**
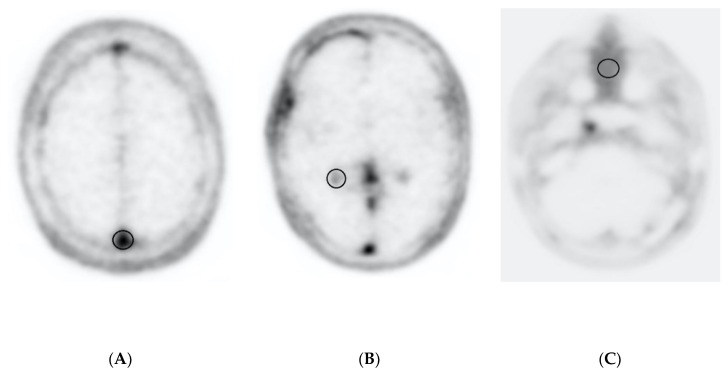
Background, region of interest. (**A**) Sagittal sinus, measured in the posterior part of the venous superior sagittal sinus; (**B**) choroid plexus, measured in the posterior horn of the right-side ventricle; (**C**) nasal mucosa.

Using syngo.via, the mean tracer activity concentration (kBq/cm^3^) of the meningiomas at each time point was measured in a manually delineated region of interest (ROI). The size of the ROI was adapted to the size of the meningioma in MRI. In detail, the ROI to determine tracer activity concentration was primarily drawn in the MRI in order to exclude physiological tracer activity concentration (i.e., pituitary gland) while at the same time providing the best possible morphological delineation of the meningioma. This ROI was transferred secondarily to the PET data set. In the case of multifocal lesions, the largest lesion was chosen. Moreover, the maximum tracer concentration of three background regions—the choroid plexus in the posterior horn of the right lateral ventricle, the venous sagittal sinus and the nasal mucosa (Figure 2)—was assessed. 

**Figure 3:**
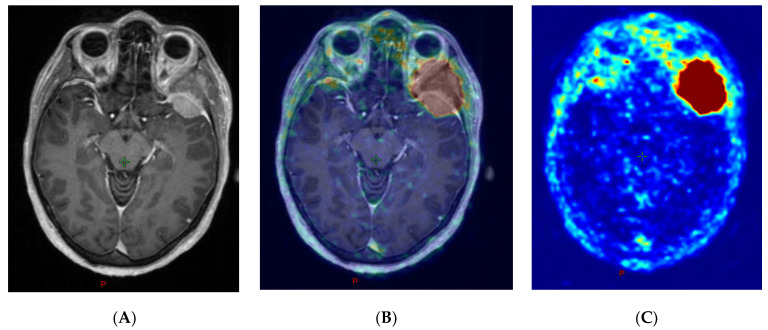
[68Ga]Ga-68-labeled [DOTA0-Phe1-Tyr3] octreotide (Ga-68-DOTATOC)-PET windowing according to MRI. (**A**) MRI, T1-weighted sequences with magnetization-prepared rapid acquisition with gradient echo; (**B**) PET windowing orientating on areas with sharp tumor demarcation in MRI using coregistered PET/MRI; (**C**) attenuation-corrected PET data.

Meningioma lesion volumes were analyzed using the radiotherapy planning suite ECLIPSE^TM^ to simulate the usage of PET data for target volume definition by the radiation oncologist. For these measurements, the window level of the PET images was adapted with the standard procedure in our department in accordance with previous studies [11]. Therefore, the lesion boundaries in fused PET and MRI images were aligned in visual inspection orientating on areas with sharp tumor demarcation such as where the tumor was adjacent to the cerebrospinal fluid (Figure 3).

PTV assessed by the radiation oncologists (10 years’ experience) was also planned by using ECLIPSE^TM^ (Version 15.5, Varian Medical Systems Inc., USA). For treatment planning, a contrast-agent-based high-resolution thin-slice (0.75 mm) CT scan was acquired. Treatment planning was based on contrast-enhanced thin-slice planning CT and a coregistered Ga-68-DOTATOC-PET/MRI dataset (T1-weighted MPRAGE, attenuation-corrected PET). The tumor volume was defined as the meningioma extension based on CT and PET/MRI datasets. PET information was especially used where tumor margins were insufficiently visualized on MRI [11]. The PTV was generated with a 0–1 mm safety margin. 

An experienced nuclear medicine physician documented the number of detected meningioma lesions on PET images alone for each patient and time point in an unblinded manner. Lesion detection rates were compared to the reference time point 60 min p.i. to identify the earliest time point with 100% lesion detection.

### 2.4. Statistical Analysis

Statistical analysis was performed using SPSS Statistics (version 25). Due to the small number of patients included in this study, a nonparametric data distribution was assumed, and descriptive parameters were generally expressed as the median and interquartile range (IQR). The Friedman test followed by a Wilcoxon signed-rank test including Bonferroni correction was used to identify the time point at which there was no further significant change in the meningiomas’ PET volume. Significance was assumed at *p* < 0.05.

## 3. Results

### 3.1. Patient Characteristics

Fifteen patients (3 males, 12 females, with a mean age of 52 years (range: 34–80)) were included in the study. Ga-68-DOTATOC-PET/MRI was performed as diagnostic imaging prior to planned RRS. Prior to PET/MRI, meningioma lesions had been treatment-naïve (four patients), partially resected (eight patients) or were followed with active surveillance (three patients). Two patients had already undergone tumor resection followed by radiotherapy prior to PET/MRI. The origin of meningiomas was either sphenoidal cavernous sinus (five patients), sphenoidal (four patients), petroclival (three patients), spheno-orbital, optic nerve sheath or temporal fossa (one patient each). According to the World Health Organization classification [14,15], most meningiomas were classified as grade I (14 patients) and only one was classified as grade II. In 12 out of 15 patients, a comparison with the documented planning target volume (PTV) of the radiation oncologists was possible. In three patients, there was no PTV due to therapy changes after PET/MRI. One patient underwent Lutetium-177-DOTATOC therapy, one patient was followed by active surveillance and one patient refused the treatment.

### 3.2. Tracer Activity Concentration Kinetics and Ratios

**Figure 4:**
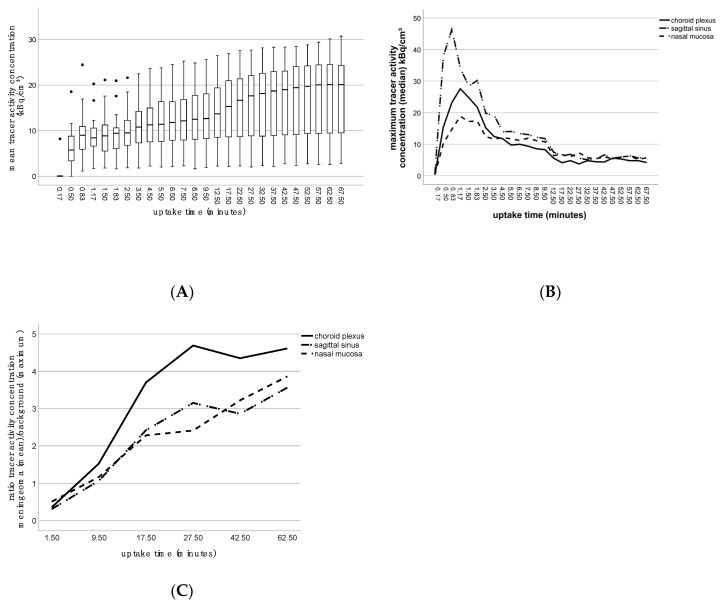
Tracer activity concentration, kinetics and ratios. (**A**) Box plots of mean tracer activity concentration (kBq/cm^3^) of all meningiomas for each time point; (**B**) line diagram showing the maximum tracer activity concentration as the median (kBq/cm^3^) of three different background regions as a function of uptake time; (**C**) line diagram depicting the median ratios of mean tracer activity concentration in meningioma lesions to maximum concentration in the backgrounds. Black dots represent the outliers.

Most lesions showed a steep initial increase of tracer activity concentration followed by a flattened curve during the dynamic 70 min examination. The mean tracer uptake reached 76.2% at 17.5 min p.i. (17.5 min: 15.3 kBq/cm^3^ (IQR, 8.6–21.1 kBq/cm^3^), 0.41 mCi/cm^3^; 62.5 min: 20.1 kBq/cm^3^ (IQR 9.2–24.6 kBq/cm^3^), 0.56 mCi/cm^3^; Figure 4A). The tracer activity concentration curves of all background regions showed a peak during the first 1.2 min p.i. (first-pass perfusion) and a continued decline of concentration thereafter. Tracer activity concentration curves of the background regions and the ratios of mean tracer activity concentrations in meningiomas to maximum concentrations in background regions are shown in Figure 4B,C. 

### 3.3. PET Volume of Meningiomas

**Figure 5:**
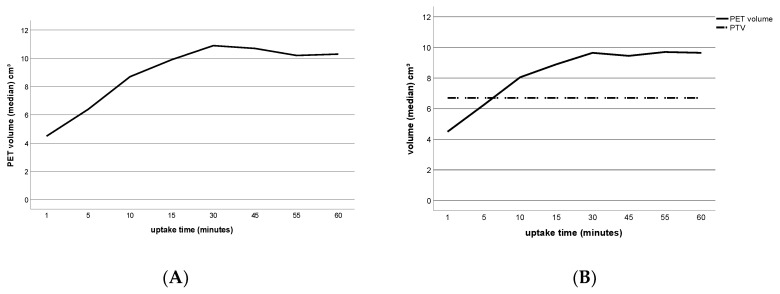
Ga-68-DOTATOC-PET volumes and corresponding planning target volume (PTV, as obtained from medical records). (**A**) Line diagram of median PET volume of all meningioma lesions as a function of uptake time; (**B**) line diagram of median PET volume as a function of uptake time (solid line) and the median PTV for comparison (dashed line). Median PET volume differs from (B) because only 12/15 patients were included due to missing PTV.

The median PET volume of all meningiomas 60 min p.i. was 10.3 cm^3^ (IQR, 6.7–14.8 cm^3^; Figure 5A). The Friedman test with the post hoc Wilcoxon signed-rank test and Bonferroni correction showed no further significant change of the PET volumes from 15 min p.i. (15 min: 9.9 cm^3^ (IQR, 6.2–15.6 cm^3^)); 60 min: 10.3 cm^3^ (IQR, 6.7–14.8 cm^3^; *p* = 0.38 without Bonferroni correction, *p* = 1.0 with Bonferroni correction). 

In 12/15 patients, a comparison with PTV documented by the referring radiation oncologist was possible (Figure 5B). The median PTV was 6.7 cm^3^ (IQR, 4.4–11.5 cm^3^). For all patients, the PET volume 60 min p.i. was larger than the PTV at the same time point with a median difference between PET volume and PTV of 2.5 cm^3^ (IQR, 1.5–3.3 cm^3^). From 10 min p.i., the PET volume exceeded the PTV in all patients.

### 3.4. Lesion Assessment

Table 1 shows the earliest time points with 100% lesion detection in each patient compared to the diagnostic standard 60 min p.i. In all patients, including cases of multifocal lesions, this was achieved from an uptake time 10 min p.i. until the end of the examination.

## 4. Discussion

**Figure 6:**
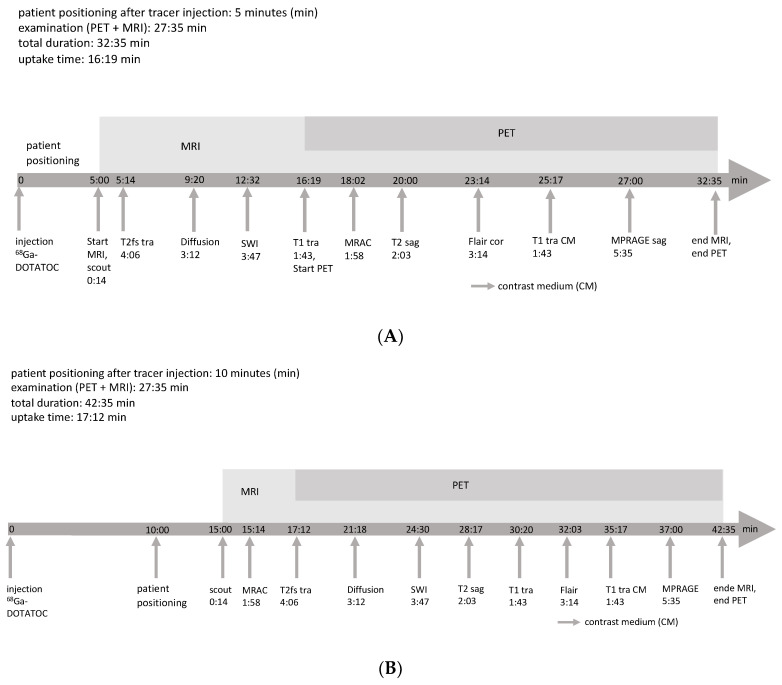
Adjusted protocol for Ga-68-DOTATOC-PET/MRI in our clinic. (**A**) Examination of the first patient of the day; (**B**) examination of the following patients. SWI, susceptibility-weighted imaging; MRAC, MRI-based attenuation correction; FLAIR, fluid-attenuated inversion recovery; MPRAGE, magnetization-prepared rapid acquisition with gradient echo; T2fs, T2-weighted sequences with fat saturation; tra, transaxial plane; sag, sagittal plane; cor, coronal plane; CM. gadolinium-based contrast medium.

Our investigation suggests that the tracer uptake time in patients with meningiomas who undergo Ga-68-DOTATOC-PET before RRS can be shortened to a minimum of 15 min after tracer injection without compromising the lesion detection rate or PET volume compared to the clinical standard uptake time of 60 min [16]. The PET volume information required for PTV delineation for RRS is not negatively influenced by the shortened tracer uptake time. The examination protocol in patients with meningiomas undergoing PET imaging before RRS was adjusted accordingly at our institution (Figure 6).

Meningiomas show high expression of the somatostatin receptor 2 [17]. Hence, PET imaging with the specific tracer Ga-68-DOTATOC can depict meningiomas with a high lesion-to-background contrast [12]. 

Sommerauer et al. showed that meningiomas with transosseous growth exhibited higher uptake compared to meningiomas with only an intracranial extent [18]. In well-differentiated meningiomas, the degree of tracer uptake correlates with tumor growth. In WHO III meningiomas, which typically show lower tracer uptake, there is no correlation with tumor growth [19]. In our study, all meningiomas were well-differentiated and hence our results cannot be directly applied to high-grade meningiomas. Yet, PET information is also very useful before radiosurgery in high-grade meningiomas, especially in cases with an intraosseous tumor extent [20]. Further studies are necessary to evaluate the dynamic tracer characteristics of high-grade meningiomas. 

Tracer uptake and kinetics depend on the WHO grade but are also likely to be influenced by prior treatment (e.g., radiation). Moreover, we assume that the type of therapy (partial resection vs. radiotherapy and Lu177-DOTATOC/TATE; i.e., cytoreductive vs. targeted) will influence tracer uptake/amplitude (i.e., maximum uptake). This means that targeted therapies would show lower maximum uptake vs. constant maximum uptake after partial resection. However, the upslope of tracer uptake over time should remain unchanged. Given the small study collective (*n* = 15) and only two patients with previous radiotherapy, we were not able to address this issue in a statistically sound manner in this study. 

Ga-68-DOTATOC-PET can significantly improve target volume delineation by helping to demarcate meningioma boundaries, especially when the lesion is adjacent to osseous structures or postoperative scar tissue [21,22]. Hybrid imaging would add value in most cases of meningiomas before RRS. Frequent use remains a challenge due to the limited number of dedicated PET devices as well as the short half-life of Ga-68-DOTATOC. The half-life of gallium-68 is 68 min [23], and the capacity of a Germanium-68/Ga-68-generator for Ga-68 synthesis is limited [24]. As a result, the number of patients who can be examined per day is restricted. Henze et al. [12] showed that the tracer activity concentration in meningiomas increases rapidly after tracer administration with high uptake as soon as 20 min p.i. and showed that, conversely, uptake of the surrounding tissue decreases rapidly without relevant tracer accumulation after 10 min p.i. [7]. Similar to these findings, the median tracer activity concentration of the meningiomas in the present study was at 76.2% at 17.5 min p.i with slightly continued Ga-68-DOTATOC accumulation thereafter, whereas background regions showed a fast washout of tracer activity concentration 1.2 min p.i. (first-pass perfusion). 

It is shown that the diagnostic performance for lesion detection in PET/MRI and PET/CT is comparable [25], and previous studies presented similar [18F]F-Flourdeoxyglucose (FDG) uptake values in PET/CT and PET/MRI [26,27]. 

In the current study, the results confirm that a shorter tracer uptake time of 15 min is feasible without detriment to diagnostic accuracy and without underestimation of the PET volume, even if the number of patients is limited.

In all assessable patients (12/15), the median PET volume after 60 min was higher than the median PTV defined by the radiation oncologists based on all available imaging data (PET/MRI after 60 min p.i. and CT). In contrast to our delineation of the raw PET volume, which included the pituitary gland when the meningioma was in contact with it, care is taken during radiation planning not to overlap PTV and organs at risk, which is why the PTV is smaller. If high radiation doses would cover the entire PET volume, the limits for risk organs and corresponding toxicities would be exceeded.

It was impossible to measure a threshold-based PET volume in the early images due to the early peak of tracer activity concentration during the first-pass perfusion. Therefore, we finally rejected threshold-based volume measurements in our study.

We used vendor-provided UTE for attenuation correction. The chosen attenuation correction (AC) can influence the tracer activity concentration, especially in skull base meningiomas, but does not significantly influence the lesion volume due to the high lesion-to-background ratio [28].

## 5. Conclusions

In patients with meningiomas undergoing Ga-68-DOTATOC-PET before RRS, the tracer uptake time can safely be reduced to 15 min p.i. with comparable PET volume and 100% lesion detection compared to 60 min p.i.

## Figures and Tables

**Table 1:** Earliest time point of 100% lesion detection compared to the standard 60 min post tracer injection (p.i).

Patient	Total Number of Lesions after 60 min p.i. (*n*)	Earliest Time Point of 100% Lesion Detection (min p.i.)
1	1	5
2	1	1
3	1	1
4	1	5
5	3	10
6	5	10
7	1	5
8	1	1
9	1	1
10	1	1
11	1	10
12	1 (diffuse)	10
13	4	10
14	2	10
15	1	1

## References

[1] Ostrom Q.T., Gittleman H., Xu J., Kromer C., Wolinsky Y., Kruchko C., Barnholtz-Sloan J.S. (2016). CBTRUS statistical report: Primary brain and other central nervous system tumors diagnosed in the United States in 2009–2013. Neuro Oncol..

[2] Rogers L., Barani I., Chamberlain M., Kaley T.J., McDermott M., Raizer J., Schiff D., Weber D.C., Wen P.Y., Vogelbaum M.A. (2015). Meningiomas: Knowledge base, treatment outcomes, and uncertainties. A RANO review. J. Neurosurg..

[3] Goldbrunner R., Minniti G.G., Preusser M., Jenkinson M.D., Sallabanda K.K., Houdart E.E., Von Deimling A.A., Stavrinou P.P., Lefranc F., Lund-Johansen M.M. (2016). EANO guidelines for the diagnosis and treatment of meningiomas. Lancet Oncol..

[4] Conti A., Pontoriero A., Midili F., Iatì G., Siragusa C., Tomasello C., La Torre D., Cardali S.M., Pergolizzi S., De Renzis C. (2015). CyberKnife multisession stereotactic radiosurgery and hypofractionated stereotactic radiotherapy for perioptic meningiomas: Intermediate-term results and radiobiological considerations. Springerplus.

[5] Di Franco R., Borzillo V., Ravo V., Falivene S., Romano F.J., Muto M., Cammarota F., Totaro G., Ametrano G., Rossetti S. (2018). Radiosurgery and stereotactic radiotherapy with cyberknife system for meningioma treatment. Neuroradiol. J..

[6] Scoccianti S., Detti B., Gadda D., Greto D., Furfaro I., Meacci F., Simontacchi G., Di Brina L., Bonomo P., Giacomelli I. (2015). Organs at risk in the brain and their dose-constraints in adults and in children: A radiation oncologist’s guide for delineation in everyday practice. Radiother. Oncol..

[7] Henze M., Schuhmacher J., Hipp P., Kowalski J., Becker D.W., Doll J., Mäcke H.R., Hofmann M., Debus J., Haberkorn U. (2001). PET imaging of somatostatin receptors using [68GA]DOTA-D-Phe1-Tyr3-Octreotide: First results in patients with meningiomas. J. Nucl. Med..

[8] Afshar-Oromieh A., Giesel F.L., Linhart H.G., Haberkorn U., Haufe S., Combs S.E., Podlesek D., Eisenhut M., Kratochwil C. (2012). Detection of cranial meningiomas: Comparison of 68Ga-DOTATOC PET/CT and contrast-enhanced MRI. Eur. J. Nucl. Med. Mol. Imaging.

[9] Laudicella R., Albano D., Annunziata S., Calabrò D., Argiroffi G., Abenavoli E., Linguanti F., Vento A., Bruno A., Alongi P. (2019). Theragnostic use of radiolabelled dota-peptides in meningioma: From clinical demand to future applications. Cancers.

[10] Galldiks N., Albert N.L., Sommerauer M., Grosu A.L., Ganswindt U., Law I., Preusser M., Le Rhun E., A Vogelbaum M., Zadeh G. (2017). PET imaging in patients with meningioma—Report of the RANO/PET Group. Neuro Oncol..

[11] Acker G., Kluge A., Lukas M., Conti A., Pasemann D., Meinert F., Nguyen P.T.A., Jelgersma C., Loebel F., Budach V. (2019). Impact of 68Ga-DOTATOC PET/MRI on robotic radiosurgery treatment planning in meningioma patients: First experiences in a single institution. Neurosurg. Focus.

[12] Henze M., Dimitrakopoulou-Strauss A., Milker-Zabel S., Schuhmacher J., Strauss L.G., Doll J., Mäcke H.R., Eisenhut M., Debus J., Haberkorn U. (2005). Characterization of 68-Ga-DOTA-D-Phe1-Tyr3-Octreotide Kinetics in Patients with Meningiomas. J. Nucl. Med..

[13] Afshar-Oromieh A., Wolf M.B., Kratochwil C., Giesel F.L., Combs S.E., Dimitrakopoulou-Strauss A., Gnirs R., Roethke M.C., Schlemmer H.P., Haberkorn U. (2015). Comparison of 68 Ga-DOTATOC-PET/CT and PET/MRI hybrid systems in patients with cranial meningioma: Initial results. Neuro Oncol..

[14] Louis D.N., Perry A., Reifenberger G., Von Deimling A., Figarella-Branger D., Cavenee W.K., Ohgaki H., Wiestler O.D., Kleihues P., Ellison D.W. (2016). The 2016 World Health Organization Classification of Tumors of the Central Nervous System: A summary. Acta Neuropathol..

[15] Kleihues P., Louis D.N., Scheithauer B.W., Rorke L.B., Reifenberger G., Burger P.C., Cavenee W.K. (2002). The WHO classification of tumors of the nervous system. J. Neuropathol. Exp. Neurol..

[16] Boy C., Poeppel T., Kotzerke J., Krause B.J., Amthauer H., Baum R.P., Buchmann I., Ezziddin S., Führer D., Gabriel M. (2018). DGN-Handlungsempfehlung (S1-Leitlinie). Somatostatin receptor PET/CT (SSTR-PET/CT). Nuklearmedizin.

[17] Graillon T., Romano D., Defilles C., Saveanu A., Mohamed A., Figarella-Branger D., Roche P.-H., Fuentes S., Chinot O., Dufour H. (2017). Octreotide therapy in meningiomas: In vitro study, clinical correlation, and literature review. J. Neurosurg..

[18] Sommerauer M., Burkhardt J.-K., Frontzek K., Rushing E., Buck A., Krayenbuehl N., Weller M., Schaefer N., Kuhn F.P. (2016). 68Gallium-DOTATATE PET in meningioma: A reliable predictor of tumor growth rate?. Neuro Oncol..

[19] Seystahl K., Stoecklein V., Schüller U., Rushing E., Nicolas G., Schäfer N., Ilhan H., Pangalu A., Weller M., Tonn J.-C. (2016). Somatostatin receptor-targeted radionuclide therapy for progressive meningioma: Benefit linked to 68Ga-DOTATATE/-TOC uptake. Neuro Oncol..

[20] Zollner B., Ganswindt U., Maihoefer C., Corradini S., Albert N.L., Schichor C., Belka C., Niyazi M. (2018). Recurrence pattern analysis after [68Ga]-DOTATATE-PET/CT -planned radiotherapy of high-grade meningiomas. Radiat Oncol. Radiat. Oncol..

[21] Milker-Zabel S., Bois A.Z.-D., Henze M., Huber P., Schulz-Ertner D., Hoess A., Haberkorn U., Debus J. (2006). Improved target volume definition for fractionated stereotactic radiotherapy in patients with intracranial meningiomas by correlation of CT, MRI, and [68Ga]-DOTATOC-PET. Int. J. Radiat. Oncol..

[22] Graf R., Nyuyki F., Steffen I.G., Michel R., Fahdt D., Wust P., Brenner W., Budach V., Wurm R., Plotkin M. (2013). Contribution of 68Ga-DOTATOC PET/CT to target volume delineation of skull base meningiomas treated with stereotactic radiation therapy. Int. J. Radiat. Oncol..

[23] Pauwels E., Cleeren F., Bormans G., Deroose C.M. (2018). Somatostatin receptor PET ligands—the next generation for clinical practice. Am. J. Nucl. Med. Mol. Imaging.

[24] Kumar K. (2020). The Current Status of the Production and Supply of Gallium-68. Cancer Biother. Radiopharm..

[25] Berzaczy D., Giraudo C., Haug A.R., Raderer M., Senn D., Karanikas G., Weber M., Mayerhoefer M.E. (2017). Whole-body 68Ga-DOTANOC PET/MRI versus 68Ga-DOTANOC PET/CT in patients with neuroendocrine tumors: A prospective study in 28 patients. Clin. Nucl. Med..

[26] Law W.P., Maggacis N., Jeavons S.J., Miles K.A. (2017). Concordance of 18F-FDG PET Uptake in Tumor and Normal Tissues on PET/MRI and PET/CT. Clin. Nucl. Med..

[27] Afaq A., Fraioli F., Sidhu H., Wan S., Punwani S., Chen S.-H., Akin O., Linch D., Ardeshna K., Lambert J. (2017). Comparison of PET/MRI with PET/CT in the evaluation of disease status in lymphoma. Clin. Nucl. Med..

[28] Rausch I., Rischka L., Ladefoged C.N., Furtner J., Fenchel M., Hahn A., Lanzenberger R., Mayerhoefer M.E., Traub-Weidinger T., Beyer T. (2017). PET/MRI for oncologic brain imaging: A comparison of standard MR-based attenuation corrections with a model-based approach for the siemens mMR PET/MR system. J. Nucl. Med..

